# Exploring Frequencies of Circulating Specific Th17 Cells against Myeloperoxidase and Proteinase 3 in ANCA Associated Vasculitis

**DOI:** 10.3390/ijms20235820

**Published:** 2019-11-20

**Authors:** Laura Martinez Valenzuela, Juliana Draibe, Maria Quero, Xavier Fulladosa, Josep Maria Cruzado, Oriol Bestard, Juan Torras

**Affiliations:** 1 Hospital Universitari de Bellvitge, Nephrology Unit. Hospitalet de Llobregat, 08907 Barcelona, Spain; lauramartinezval252@gmail.com (L.M.V.); jbordignon@bellvitgehospital.cat (J.D.); m.quero@bellvitgehospital.cat (M.Q.); xfulladosa@bellvitgehospital.cat (X.F.); jmcruzado@bellvitgehospital.cat (J.M.C.); obestard@bellvitgehospital.cat (O.B.); 2 IDIBELL Institut d’Investigacions Biomèdiques, Hospitalet de Llobregat, 08907 Barcelona, Spain; 3 Facultat de Medicina, Campus de Bellvitge, Universitat de Barcelona. Hospitalet de Llobregat, 08907 Barcelona, Spain

**Keywords:** ANCA, vasculitis, Th17, IL-17, immunology, lymphocyte, biomarker

## Abstract

Background: The role of the T helper 17 (Th17) cell subset in anti-neutrophil cytoplasm antibodies (ANCA) associated vasculitis (AAV) is controversial. We hypothesized that a specific Th17 response to myeloperoxidase (MPO) or proteinase 3 (PR3) is detectable in AAV patients and is different among the disease phases. Methods: We analyzed 43 AAV patients with renal involvement (21 acute and 22 remission patients), and 12 healthy controls. Peripheral blood mononuclear cells (PBMCs) were cultured with PR3/MPO over 48 h. Thereafter, frequencies of MPO/PR3-specific Th17 cells were assessed using an enzyme-linked immunosorbent spot (ELISpot) assay. Supernatant IL-17 concentration was quantified using ELISA. Finally, specific Th17 response after depletion of T regulatory lymphocytes (T-regs) in some remission patients was compared to the non T-reg-depleted response. Results: Specific Th17 cell number was higher in acute patients compared to remission (*p* = 0.004). Specific Th17 cell number performed well in the disease activity detection (ROC curve area under the curve (AUC) = 0.87; *p* = 0.0001) with an optimal cut-off of 6 spots/million. Patients above this cut-off showed higher serum creatinine (*p* = 0.004), C-reactive protein (CRP) (*p* = 0.001) and ANCA titer (*p* = 0.032). Supernatant IL-17 concentration was higher in acute patients compared to remission (*p* = 0.035) and did not normalize to healthy control levels (*p* = 0.01). Conclusions: A specific Th17 cell response is present in AAV patients. This response is more pronounced in the acute phase, but persists in remission.

## 1. Introduction

Anti-neutrophil cytoplasm antibodies (ANCA) associated vasculitis (AAV) is an autoimmune multisystemic disease that causes fast and severe kidney impairment. Glomerulonephritis in AAV is pauciimmune. ANCA are directed against myeloperoxidase (MPO) and proteinase 3 (PR3) inside neutrophil granules that are exposed in the cell membrane after a priming stimulus [1]. ANCA causes neutrophil degranulation, endothelial damage, and leads to an inflammatory response [2]. In the recent years, important attention has been focused on adaptive immunity, and particularly on T lymphocytes as main effectors and amplifiers of the autoimmune response in AAV [3].

The implication of the T helper 17 (Th17) lymphocyte subset has been described in several autoimmune diseases [4], such as rheumatoid arthritis [5], systemic lupus erythematosus [6], or other systemic vasculitides [7,8]. Th17 cells produce proinflammatory cytokines pertaining to the interleukin-17 (IL-17) family [9]. Interest in the Th17 subset in AAV is due to the close interplay between neutrophils and Th17 cells. Th17 cells enhance recruitment of neutrophils while neutrophils induce chemotaxis of Th17 cells [10]. 

Th17 response in AAV has been previously studied with controversial findings. Some authors reported a higher serum IL-17 concentration in AAV patients [11] compared to healthy controls, thus inferring a higher number of circulating Th17 lymphocytes [12]. Other authors found a higher total number of circulating Th17 cells in AAV patients compared to healthy controls without differences between disease phases [12]. Conversely, other authors did not find any expansion of the Th17 response [13,14]. The frequency of specific auto-reactive Th17 cells responding to PR3 or MPO has been assessed only in remission patients and found to be higher compared to healthy controls [11,15]. The Th17 response is suppressed by T regulatory lymphocytes (T-regs) in the absence of disease [16]. In contrast, T-regs from AAV patients have impaired inhibitory ability [17] and even can stimulate Th17 response [18].

The specificity of the current tools to assess disease activity, such as C-reactive protein (CRP) [19], ANCA titer [20], and hematuria [21], is still challenging. Therefore, a better understanding of the pathophysiology of AAV and the development of new biomarkers is crucial to identify in patients with persistent disease activity, in order to individualize the immunosuppressive treatment and minimize relapses and adverse events. 

Given the debated data concerning the Th17 subset, in the present study we aimed to comprehensively characterize and compare the response of this T-cell subset (serum, urine, and cell kinetics after MPO/PR3 stimulation) in AAV patients in the different phases of the disease. 

## 2. Results

### 2.1. Baseline Characteristics of the Population

Forty-six AAV patients were eligible for the study. Of those, 43 AAV patients (21 in the acute phase and 22 in the remission phase of the disease) accomplished inclusion and exclusion criteria. Of the 21 acute patients, 16 were recruited at the moment of the diagnostic. The remaining 5 were on a disease flare. We also included 12 healthy controls.

Baseline characteristics of the population are shown in Table 1. Mean follow-up of the remission cohort was 27.64 months. Acute patients showed higher serum creatinine and proteinuria compared to patients of the remission cohort (*p* = 0.041 and *p* = 0.015). Anti-MPO was the most frequent specificity of the ANCA antibodies in the acute and the remission cohort. The diagnostic kidney biopsies were similar in both cohorts. Most patients in both cohorts were on the sclerotic or mixed categories of the Berden histopathologic classification (35 and 30% of patients respectively in the acute cohort and 28.6 and 23.8% in the remission cohort). Twenty percent of patients in the acute cohort were in the sclerotic category of this classification, similar to the 33.3% of patients in the remission cohort. 

Healthy controls in our study were individuals without any known medical condition, and they were not under any pharmacological treatment. Table 2 shows baseline characteristics of the healthy controls.

### 2.2. IL-17 Concentration in Serum and Urine Samples

Concentration of IL-17 in serum and urine was not different between patients in the acute or the remission phases and healthy controls. Mean serum IL-17 was 33.80 ± 60.64 ng/mL in the acute cohort, 50.52 ± 48.37 ng/mL in the remission cohort (*p* = 0.35) and 29.68 ± 35.46 ng/mL in healthy controls (ANOVA *p* = 0.3). Urinary IL-17 was 4.49 ± 4.42 ng/mL in the acute cohort, 5.67 ± 4.13 ng/mL in the remission cohort (*p* = 0.46) and 4.77 ± 3.86 ng/mL in healthy controls (ANOVA *p* = 0.68).

Focusing in the acute cohort, patients within the sclerotic or mixed classes in the Berden classification showed lower urinary IL-17 compared to focal or crescentic class (4.13 ± 4.59 vs. 8.15 ± 0.35; *p* = 0.043) with no differences in serum IL-17. Urinary IL-17 did not correlate with serum creatinine (*p* = 0.29).

We did not find differences in the urinary and serum IL-17 regarding the rest of the main clinical variables analyzed.

### 2.3. Circulating MPO-Specific and PR3-Specific Th17 Frequencies, Supernatant IL-17 Concentration and AAV Disease Phase

**Figure 1:**
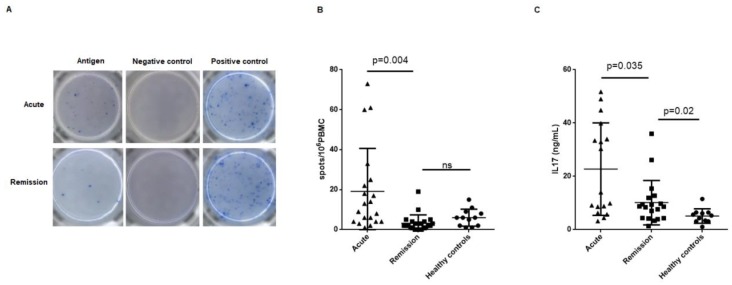
Th17 response to the stimulation with MPO or PR3. (**A**) Representative wells of an enzyme-linked immunosorbent spot (ELISpot) plate corresponding to an acute and a remission phase patient. Antigen wells contain PBMCs stimulated with proteinase 3 or myeloperoxidase. Negative control wells contain PBMCs cultured with medium alone. Positive control wells contain PBMCs stimulated with phytohemagglutinin. (**B**) Number of specific IL-17 producing cells in response to MPO or PR3 in the ELISpot assay. Number of spots/10^6^PBMCs was significantly higher in the acute phase patients, and lowered in remission to healthy control level. (**C**) Concentration of IL-17 present in the supernatant of PBMCs culture after stimulation with MPO or PR3 over 48 h. Supernatant IL-17 concentration was higher in acute patients compared to remission patients. Supernatant IL-17 concentration did not normalize in remission. Th17—T helper 17; MPO—myeloperoxidase; PR3—proteinase 3; AAV—anti-neutrophil cytoplasm antibodies associated vasculitis; PBMCs—peripheral blood mononuclear cells; IL-17—interleukin-17.

Circulating MPO and PR3-specific Th17 frequencies assessed by enzyme-linked immunosorbent spot (ELISpot) were different between acute and remission AAV patients. Figure 1A shows representative wells of an ELISpot plate corresponding to an acute and a remission phase patient.

The frequency of the PR3/MPO-specific IL-17-producing T cells was higher in acute patients compared to remission patients (19.15 ± 21.49 vs. 3.18 ± 4.20 spots/10^6^ PBMCs; *p* = 0.004), which were similar to the healthy controls (6 ± 4.28 spots/10^6^ PBMCs) (Figure 1B).

IL-17 supernatant concentration of peripheral blood mononuclear cells (PBMCs) cultures stimulated with MPO/PR3 antigens was higher in acute patients compared to remission patients (19.35 ± 17.32 vs. 9.91 ± 8.55 ng/mL; *p* = 0.035). Supernatant IL-17 remained higher in remission patients compared to healthy controls (9.91 ± 8.55 vs. 5.12 ± 2.72; *p* = 0.02) (Figure 1C). 

**Figure 2:**
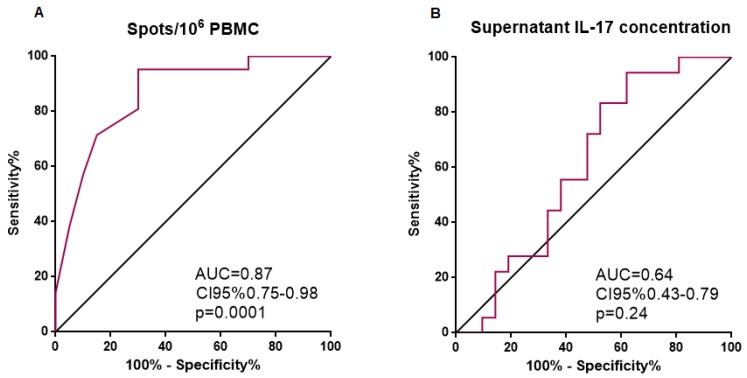
Receiver operator curves of number of spots/10^6^ PBMCs and supernatant IL-17 concentration. (**A**) Number of spots/10^6^ PBMCs showed a good performance as a disease activity biomarker, as reflected by AUC = 0.87 (CI 95%: 0.75–0.98). (**B**) Supernatant IL-17 did not perform well as a disease activity biomarker. PBMCs—peripheral blood mononuclear cells; IL-17—interleukin-17; AUC—area under the curve; CI—confidence interval.

We plotted a ROC curve in order to evaluate the classificatory performance of the frequencies of Th17 cells and supernatant IL-17 concentrations between acute and remission patients. Number of spots/10^6^ PBMCs showed an area under the curve (AUC) = 0.87 (*p* = 0.0001) for the detection of disease activity, with a 70% sensitivity, 95.45% specificity, and likelihood ratio = 15.40 for a cut-off of 6 spots/10^6^ PBMCs in our AAV patient cohort (Figure 2). On the contrary, supernatant IL-17 showed an AUC = 0.61 (*p* = 0.24).

**Figure 3:**
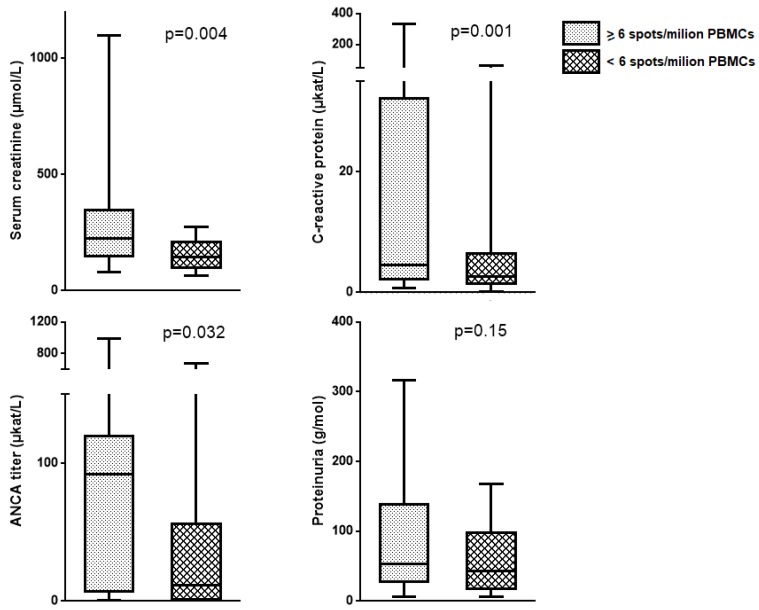
Comparison of clinical parameters among the two groups of AAV patients defined by the cut-off of 6 spots/10^6^ PBMCs. Patients above this cut-off showed higher serum creatinine, ANCA titer, and CRP levels compared to the rest. We did not find differences regarding to proteinuria. ANCA—anti-neutrophil cytoplasm antibodies; CRP—C-reactive protein.

We divided the whole cohort of AAV patients into two groups defined by the optimal cut-off of 6 spots/10^6^ PBMCs. Thereafter, we compared the clinical variables among the two groups. We found higher serum creatinine, CRP level, and ANCA titer (*p* = 0.032) in patients with ≥6 spots/10^6^ PBMCs compared to the rest. We did not find differences regarding the proteinuria (Figure 3).

We performed a univariate binary logistic regression analysis evaluating most relevant variables predicting disease activity. Of those, only the MPO/PR3-specific Th17 frequencies and supernatant IL-17 concentration were shown to be disease activity predictors. Thereafter, we constructed a multivariate model including those variables with a *p*-value < 0.1 in the univariate analysis. Only specific Th17 frequencies and supernatant IL-17 concentration were independent predictors of disease activity in our cohort (Table 3).

Patients included in the remission cohort that later presented a relapse during the follow-up period of the study (18.1%) showed similar values of Th17 specific lymphocytes and IL-17 supernatant concentration compared to those remission patients who did not relapse (*p*-values = 0.82 and 0.28, respectively). 

### 2.4. T Regulatory Lymphocytes do not Suppress Th17 Response to MPO in AAV Patients

**Figure 4:**
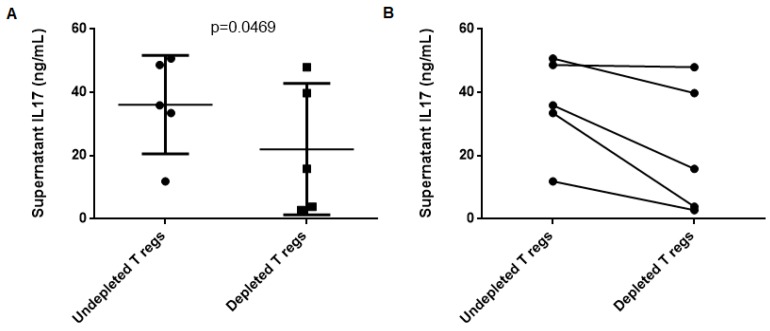
Change in IL-17 production by PBMCs in response to MPO after T-regs depletion in 5 remission patients. T-regs depleted PBMCs produced less IL-17 in response to MPO or PR3 compared to PBMCs without T-regs depletion. PBMCs—peripheral blood mononuclear cells; IL-17—interleukin-17; T-regs—T regulatory lymphocyte; MPO—myeloperoxidase.

Finally, we aimed to determine if T-regs played a certain role modulating the specific Th17 response found during AAV remission. After depleting T-regs from PBMC in 5 patients showing stable remission, T-reg-depleted PBMC samples showed less IL-17 production in response to MPO compared to the non-depleted PBMCs from the same patient (*p*-value = 0.0469) (Figure 4).

## 3. Discussion

Results obtained in this study support a role for the Th17 cell subset in AAV pathogenesis. Some other authors previously demonstrated higher total number of circulating Th17 cells in AAV patients compared to healthy controls, with no differences between the acute and the remission phases of the disease [12,22]. Nevertheless, few authors have reported the specificity of Th17 cells against MPO or PR3, and only in the remission phase. Thus, a previous study evaluated IL-17 specific response to ANCA antigens by ELISpot in remission AAV patients compared to healthy controls [11]. They found presence of specific Th17 cells in 53% of the remission patients compared to none of the healthy controls. Abdulahad et al. also found a higher specific IL-17 responsiveness to PR3 stimulation by flow cytometry in AAV remission patients compared to healthy controls, but they did not study patients in the acute phase [15]. The majority of AAV patients in our study showed detectable circulating specific MPO/PR3 IL-17-producing cells in the ELISpot test. To our knowledge, this is the first study that shows a significantly higher Th17 responsiveness to MPO/PR3 in the acute phase compared to the remission phase of the disease. The results of the analysis of supernatant IL-17 concentration are in agreement with those obtained in the ELISpot assay, with a higher amount of IL-17 produced in response to MPO/PR3 in acute patients. Interestingly, higher IL-17 production persisted even in remission compared to healthy controls.

Regarding the performance of the specific Th17 cells expansion parameters as disease activity biomarkers, specific Th17 frequencies retrieved the best results. The number of specific Th17 spots/10^6^ PBMCs showed a good performance as a biomarker of disease activity. After comparing the value of classical surrogate markers of disease activity in AAV patients, our results suggest the utility of the 6 spots/10^6^ PBMCs cut-off to detect such activity. Patients above this cut-off presented with worse kidney function, higher ANCA titer, and more elevated CRP levels. Interestingly, differences in ANCA titer and CRP levels were not noticed when we divided the cohort of AAV individuals into acute and remission patients according to BVAS, but appeared when we applied the 6 Th17 specific spots/million PBMCs cut-off. These results suggest that the measure of the Th17 specific response may be complementary to the classical biomarkers in the evaluation of the AAV activity in the clinical practice.

Serum and urinary IL-17 levels were not different in AAV patients in our cohort compared to healthy controls. Although other authors found higher levels of serum IL-17 in AAV patients compared to healthy controls [11], some other authors did not find such differences [12,13] in line with our results. The absence of differences in serum IL-17 may be due to a hypothetical localization of the Th17 response in the damaged tissues or a different sensitivity of the test used. In addition, Krohn et al. found higher serum levels of IL-17-C in AAV patients compared to healthy controls with no differences in IL-17-A [23]. Thus, the absence of differences in IL-17-A serum levels in our cohort of patients does not definitively exclude the participation of the Th17 subset in AAV [24].

Urine IL-17 levels in AAV patients have not been previously reported in the literature. In other glomerulonephritides, it has been proposed that urinary IL-17 reflects disease activity [25]. Velden et al. demonstrated the presence of IL-17 producing cells in kidney biopsies from acute AAV patients, with a positive correlation between the number of Th17 cells and serum creatinine [26]. Interestingly, in our cohort of acute patients, those with more inflammatory histologies according to the Berden classification (focal and crescentic classes) showed significantly higher urinary IL-17 compared to the rest of histologies that exhibit more fibrotic lesions and less cellular infiltrates. Thus, we hypothesize that urinary IL-17 is shedded to the urinary space by these Th17 cells infiltrating the kidney in AAV, and may reflect the extent of the of Th17 cellular infiltrates.

T-regs do not suppress the specific Th17 response to MPO in our cohort of AAV patients. Surprisingly, we observed a decrease in IL-17 secretion after the depletion of the T-regs. Abdulahad et al. described a functional defect in T-regs from AAV patients, and they noticed that T-regs from some AAV patients induced increased proliferation of effector T cells [18]. The same authors recently found a skewing towards a different T-reg phenotype in AAV patients. This T-reg phenotype releases IL-17 and also stimulates IL-17 production and proliferation of the Th17 cells [27]. Rimbert et al. found a trend towards a lower IL-17 production in response to a polyclonal stimulation when PBMCs were T-reg-depleted [14]. In line with those authors findings, we observed that T-regs seem to enhance Th17 responsiveness to MPO, given the fact that IL-17 production was lower after depleting PBMCs of T-regs.

Treatment with secukinumab (recombinant monoclonal antibody against IL-17) is being investigated in other autoimmune conditions, such as psoriasis [28], but no current studies are being conducted in vasculitis. Our results provide a rational for considering IL-17 as a possible therapeutical target in AAV patients.

The main strengths of the study are: The first is the evaluation of the response against specific AAV antigens; second is the finding of a differential specific response according to the disease phase not noticed previously in other AAV cohorts; and third is being the largest cohort of AAV patients evaluated using an ELISpot assay. The main limitation is the lack of an independent validation cohort.

## 4. Methods

### 4.1. Experimental Design and Study Population

This is an observational prospective study. All patients diagnosed and prospectively followed at the Bellvitge University Hospital between January 2015 and December 2018 with renal involvement of the disease were eligible for the study. AAV diagnosis was made according to compatible clinical signs and symptoms, serum ANCA positivity, and corresponding histological lesions. All patients had renal involvement of the disease assessed by kidney biopsy. Patients were classified in two groups, patients in the acute phase of the disease and patients in the remission phase. Disease activity, as relapse, was defined as the appearance of 1/24 items of the Birmingham Vasculitis Activity Score [29] (BVAS) indicating threatened function of a vital organ attributable to vasculitis or appearance of at least three other BVAS items. Remission of the disease was defined as a BVAS of 0. Induction to remission treatment consisted on plasma exchange or intravenous metilprednisolone pulses followed by rituximab or oral/intravenous cyclophosphamide. Maintenance treatment consisted of oral prednisone (2.5–5 mg daily), and/or mycophenolate mofetil (500 mg twice a day), or mycophenolic acid (360 mg twice a day) as supported by the EULAR/ERA-EDTA guidelines [30]. Exclusion criteria were as follows, dialysis dependence, presence of infection, added autoimmune conditions, or neoplasms at the moment of the inclusion. All patients signed informed consent prior to the inclusion to the study. The Ethical Committee of Bellvitge University Hospital approved the study protocol (PR096/17) on 22 June 2017.

### 4.2. Clinical Variables

We recorded main demographical, clinical, and analytical variables, such as age, sex, time since diagnosis or relapse, history of relapse, extra-renal involvement, renal histology according to the Berden Histopathologic classification [31], ANCA specificity and titer (Wieslab^®^ Capture MPO-ANCA test kit and Wieslab^®^ Capture PR3-ANCA test kit), creatinine, proteinuria, hematuria, CRP, and the prospective evolution of those parameters during follow-up.

### 4.3. Sampling and Laboratory Procedures

We obtained peripheral blood and urine samples. We isolated peripheral blood mononuclear cells (PBMC) by Ficoll–Paque density gradient. Th17 response was assessed by: 

(1) Serum and urinary IL-17 levels using an enzyme-linked immunoassay (ELISA) assay; 

(2) Circulating frequencies of IL-17-producing T cells after stimulation with MPO or PR3 antigens using an enzyme-linked immunosorbent spot (ELISpot) platform; 

(3) IL-17 concentration in supernatants of PBMCs cultures stimulated with MPO/PR3. 

### 4.4. IL-17 Levels in Urine, Serum, and Cell Culture Supernatant

We determined IL-17-A concentration by ELISA using a commercial kit (Human IL-17-A, Thermo Fisher Scientific^®^) according to manufacturer’s instructions.

### 4.5. Circulating Th17 Cell Frequencies

We determined Th17 cell frequencies by ELISpot using a commercial kit (Human IL-17 Single-Color ELISpot, Immunospot^®^). Briefly, we cultured PBMCs for 48 h at a concentration of 10^6^ PBMC/400 µL medium (HyClone^®^ RPMI-1640 medium) under the following 3 conditions, medium alone (negative control), phytohemagglutinin (PHA) 30 µg/mL (positive control), and MPO (Human Leukocyte Myeloperoxidase, Merk^®^) or PR3 (Human non recombinant Proteinase 3, Diarect^®^) 10 µg/mL. After stimulation, PBMCs were washed twice in medium alone, and 500000 cells per well were plated in duplicate for every condition. The detection of the spots was performed after 24 h using an AID^®^ ELISpot plate reader (4th generation). Mean IL-17 spots/10^6^ PBMCs of the two well duplicates were used in the statistical analysis.

### 4.6. MPO-Specific Functional Suppressive Activity of Regulatory T cells

Five patients in stable remission were further assessed for T-reg suppressive activity on Th17 cells. PBMCs were cultured with MPO for 48 h and supernatant IL-17 levels were determined as previously described. In addition, PBMCs from the same individuals were depleted of T-regs using fluorescence activated cell sorting (FACS). T-regs were identified as CD3+, CD4+, CD25^hi^, and CD127^low^cells (anti CD3 APC Vio770, anti CD4 FITC, anti CD25 APC, anti CD127PE Vio770 antibodies, MiltenyiBiotec^®^). Briefly, PBMCs concentration was adjusted to 106 cells/100µL of sorting buffer, and incubated with 2 µL of antibody/million PBMCs during 10 min. Cell sorting was performed using a MoFloAstrios Cell Sorter. After T-regs were discarded, cell culture with the same MPO stimulation was subsequently carried out using the remaining PBMCs, and supernatant IL-17 was determined.

### 4.7. Statistical Analysis

Data was analyzed using GraphPad Prism version 6.00 (GraphPad Software, La Jolla California USA) and IBM SPSS Statistics Version 20.0 (IBM corp., Armonk, NY, USA). Kolmogorov–Smirnov test was applied to determine whether quantitative variables were normally distributed. For comparison of means of the two groups, Student’s T or Mann–Whitney U was used depending on the distribution of the variable. For comparison of means among more than two groups, ANOVA or Kruskall–Wallis test was used depending on the distribution of the variable. Chi-square test was used for qualitative variables. Correlations were assessed using Spearman’s or Pearson correlation. ROC curves were plotted. Uni- and multivariate logistic regression was used to assess if measured variables were associated with the observed outcomes. *p*-values under 0.05 were considered significant.

## 5. Conclusions

Herein we show the expansion of the Th17 cell subset in the AAV patients. We demonstrated a higher responsiveness of this lymphocyte subset to MPO and PR3 in AAV when compared to healthy controls, as well as a higher absolute number of circulating specific Th17 cells in the acute patients compared to remission patients. Higher Th17 responsiveness pointed towards persistent disease activity in our cohort and it may be explored as a part of the patient evaluation together with the classic biomarkers or a possible future therapeutic target.

## Figures and Tables

**Table 1:** Demographic characteristics of the AAV patients.

	Acute (*n* = 21)	Remission (*n* = 22)	*p*-Value
Male/Female Sex (%)	47.6/52.3	40.1/59.9	0.658
Age (years)	61.52 ± 20.23	65.81 ± 13.60	0.417
BMI	30.79 ± 6.85	27.17 ± 6.43	0.18
Time since diagnostic (months)		62.86 ± 48.43	
Recently diagnosed patients Patients in flare	0 ± 0 100.60 ± 90.84		0.001 0.20
Creatinine (µmol/L)	280.19 ± 248.99	158.95 ± 60.13	0.041
Proteinuria (g/mol)	97.13 ± 79.36	46.29 ± 39.55	0.015
ANCA titer (karb.u./L)	228.4 ± 382.28	76.66 ± 161.84	0.104
ANCA specificity (%)			1
MPO	76.2	72.7	
PR3	23.8	27.3	
CRP (µkat/L)	42.90 ± 79.95	6.09 ± 14.94	0.056
Hematuria (%)	76.2	59	0.232
History of prior relapse	-	40.9	
Lung involvement (%)	28.6	31.8	0.817

Statistically significant differences were only observed regarding creatinine and proteinuria levels between the two cohorts. ANCA—anti-neutrophil cytoplasm antibodies; MPO—myeloperoxidase; PR3—proteinase 3; CRP—C-reactive protein. BMI—body mass index.

**Table 2:** Baseline characteristics of healthy controls.

	Healthy Controls (*n* = 12)
Male/Female Sex (%)	41.66/58.33
Age (years)	36 ± 11.28
Body Mass Index	26.58 ± 2.24

**Table 3:** Uni- and multivariate analysis.

**Univariate Analysys**			
**Variable**	**OR**	**CI (95%)**	***p*-Value**
Specific Th17 frequency	1.233	1.048–1.452	0.012 *
Serum IL-17	0.993	0.979–1.008	0.377
Urinary IL-17	0.732	0.488–1.097	0.13
Supernatant IL-17	1.076	1.012–1.144	0.02 *
Serum creatinine	1.007	1–1.014	0.065
GFR	1.002	0.99–1.013	0.785
ANCA titer	1.002	0.999–1.006	0.14
CRP	1.003	0.999–1.68	0.09
**Multivariate Analysis**			
**Variable**	**OR**	**CI (95%)**	***p*-Value**
Specific Th17 frequency	1.183	1.006–1.390	0.042 *
Supernatant IL-17	1.079	1.004–1.160	0.04 *
Serum Creatinine	0.999	0.986–1.013	0.939
CRP	1.021	0.980–1.064	0.313

Uni- and multivariate binary logistic regression revealed MPO/PR3-specific Th17 frequencies and supernatant IL-17 concentration as independent predictors of AAV disease activity. OR—odds ratio; CI—confidence interval; IL-17—interleukin-17; GFR—glomerular filtration rate; ANCA—anti-neutrophil cytoplasm antibodies; CRP—C-reactive protein. * *p* value < 0.05.
